# Availability, utilization and quality of emergency obstetric care services in Sousse, Tunisia

**DOI:** 10.11604/pamj.2021.38.272.17758

**Published:** 2021-03-16

**Authors:** Manel Limam, Faten Hachani, Mariem El Ghardallou, Mouadh Bachraoui, Manel Mellouli, Ali Mtiraoui, Hedi Khairi, Thouraya Ajmi, Chekib Zedini

**Affiliations:** 1 University of Sousse, Faculty of Medicine of Sousse, Department of Family and Community Medicine, Research Laboratory “LR12ES03”, 4000, Sousse, Tunisia,; 2 University of Sousse, Faculty of Medicine of Sousse, Research Laboratory “LR12ES03”, Farhat Hached Hospital, Gynaecology and Obstetrics Department, 4000, Sousse, Tunisia

**Keywords:** Emergency obstetric care, maternal health, Sousse, Tunisia

## Abstract

**Introduction:**

emergency obstetric care (EmOC) is a high-impact priority intervention strongly recommended for improving maternal health outcomes. The objectives of this study were to assess the availability, utilization, and quality of emergency obstetric care services in the Governorate of Sousse (Tunisia).

**Methods:**

a cross-sectional study was conducted among public health facilities which performed deliveries in Sousse in 2017. Data were collected by consulting clinical records and registers and interviewing staff using WHO EmOC tools. Emergency obstetric care (EmOC) indicators were calculated.

**Results:**

only the University maternity Unit functioned as full comprehensive EmOC facility. No other public facility provided all the 7 Basic EmOC signal functions 3 months prior to the survey. The unperformed signal functions were: administration of parenteral antibiotics, manual removal of placenta and assisted vaginal delivery. The number of EmOC facilities was 0.72 per 500,000 inhabitants. The met need for EmOC was 89.5%. The proportion of caesarean section was 24.2%. The direct obstetric case fatality rate was 0.159% and intrapartum and very early neonatal death rate was 0.65%.

**Conclusion:**

raising maternity facilities to a minimum level of basic EmOC status would be a major contributing step towards maternal mortality reduction.

## Introduction

While most pregnancies and births are uneventful, all pregnancies are at risk. Around 15% of all pregnant women develop a potentially life-threatening complication that calls for skilled care, and some will require a major obstetrical intervention to survive [1,2]. It was estimated that in 2015, roughly 303000 women died during and following pregnancy and childbirth. Almost all maternal deaths (99%) occur in developing countries [3-5]. Improving maternal health was one of the eight millennium development goals adopted by the international community in 2000. Then it continues to be one target under sustainable development goal which is to reduce the global maternal mortality ratio (MMR) to less than 70/100000 births [3]. The vast majority of maternal deaths (70%) are due to direct obstetric complications: haemorrhage, sepsis, complications of abortion, hypertensive disorders of pregnancy, prolonged/ obstructed labour, ruptured uterus and ectopic pregnancy [6].

In Tunisia, nationally, the MMR is still relatively high. It was estimated to 44.8/100000 live births with regional disparities and extremes ranging from 27.9 to 67/100000 live births [7]. Low MMR in the West is due today, essentially, to the fact that obstetric complications are identified and treated promptly with a well-functioning health system. Programs to reduce maternal mortality in resource-poor settings, where MMR are high, must be able to treat these complications. In fact, Emergency obstetric care (EmOC), access to family planning and skilled assisted deliveries are three key interventions that have been implemented globally to reduce maternal mortality [8]. The EmOC was first described and internationally agreed upon in 1997 by WHO, UNICEF and UNFPA. It consists of key interventions (or signal functions) that must be available at health-care facilities designated to provide either comprehensive or basic EmOC functions [1,9]. A set of EmOC process indicators, also known as the United Nations (UN) process indicators, was also developed [9]. These indicators has been used in many countries to estimate the availability, accessibility, utilization, and quality of EmOC services, to monitor and evaluate the impact of interventions improving maternal and child health outcomes [10]. The aim of this study was to assess the availability, utilization, and quality of EmOC services in the Governorate of Sousse (Tunisia).

## Methods

**Study design and study population:** a cross-sectional study was conducted at health facility level from May 2017 to July 2017 in the governorate of Sousse (Tunisia). Data were obtained from all public health facilities which performed deliveries in the 12 months prior to the study in Sousse (5 maternity facilities: Farhat Hached University hospital, Msaken, Kalaa Kebira, Enfidha and Bouficha).

**Data collection tools, EmOC functions and indicators:** data were collected using structured, pre-established EmOC tools developed by AMDD (Averting Maternal Death and Disability) and the United Nations agencies, namely WHO, UNICEF, and UNFPA [1]. These tools are based on the EmOC indicators specified in the international guidelines for monitoring the availability and use of obstetric and neonatal services. The first instrument is the demographic module, which captures regional level data such as crude birth rate (CBR) and total population by region, etc. The second was designed to capture the performance of signal functions, described in monitoring emergency obstetric care: a handbook [1], in the last three months through interviews with heads of units and principles health workers. Finally, the third one was a summary of service statistics for the facility over 12 consecutive months. The items counted were vaginal and caesarean deliveries, direct and indirect obstetric complications, direct and indirect maternal deaths, and the number of stillbirths and very early neonatal deaths (before 24 hours). A Basic Emergency Obstetric Care (BEmOC) facility performs all of the following 7 signal functions: 1) administration of injectable antibiotics; 2) administration of oxytocic drugs; 3) administration of anticonvulsants; 4) manual removal of the placenta; 5) removal of retained products; 6) assisted vaginal deliveries; and 7) neonatal resuscitation. A comprehensive Emergency Obstetric Care (CEmOC) facility performs all signal functions in BEmOC as well as caesarean sections and blood transfusions. The eight UN process indicators are calculated and their benchmarks are presented in Table 1 [1,10]. In addition to obtaining data through individual interviews with the facility health workers, we extracted data from the facilities´ records, including registers of labour and delivery, the operating room and the antenatal and gynaecological ward. Data on maternal complications and deaths at each facility were collected retrospectively on a monthly basis for 12 months (January 2016 to December 2016) for the peripheral and regional maternities (Msaken, Kalaa Kebira, Enfidha and Bouficha) as for the maternity of the University Hospital, a sample of 4 months was selected (Year 2016) because the number of deliveries exceeded 10000 this year.

**Table 1:** emergency obstetric care indicators description

EmOC indicators	Description	Acceptable level
Availability of EmOC: BEmOC and CEmOC facilities	Ratio of EmOC facilities to the population	≥ 5 EmOC facilities per 500000 inhabitants ≥ 1 CEmOC facilities per 500000 inhabitants
Geographic distribution of EmOC facilities	Ratio of EmOC facilities at subnational level	As above
Proportion of all births performed in EmOC facilities	Proportion of all births in the population in EmOC facilities	Recommended level to be set locally
Met need for EmOC	Proportion of women with major direct obstetric complications treated in EmOC facilities	100%
Caesarean delivery as a proportion of all births	Proportion of all births in the population by caesarean delivery in EmOC facilities	5-15%
Direct obstetric case fatality rate	Proportion of women with major direct obstetric complications who die in EmOC facilities	< 1%
Intrapartum and very early neonatal death rate	Proportion of births that result in an intrapartum or very early neonatal death (<24 h) in EmOC facilities	To be determined
Proportion of maternal deaths with indirect causes	Percentage of all maternal deaths in EmOC facilities with indirect causes	None set

**Statistical analysis:** to determine indicators, we first had to estimate the total number of expected births and direct complications in the region, using the CBR and the total population. Total population figures and CBR were estimated by projection with data from the national census carried out in 2014. The number of expected direct obstetric complications used to estimate the need for EmOC was obtained by multiplying the expected number of births by 15%, based on the fact that all pregnancies are risky but 15% of them are expected to experience a direct complication. Formulas for calculating indicators are described in the AMDD documentation. Descriptive statistics were used to present our results.

**Ethical considerations:** an informed consent was obtained from the regional health directorate, health facilities heads and interviewed staff members. All data were processed with strict confidentiality.

## Results

**Availability of EmOC services:** only the maternity of the university hospital functioned as full CEmOC facility; it performed all 9 signal functions in the 3-month period preceding the survey (Table 2). The number of CEmOC facilities was 0.72 per 500000 inhabitants (Table 3). Among the rest of facilities (n=04), none provided all the 7 BEmOC signal functions 3 months prior to the survey (Table 2). None of peripheral maternities performed: administration of parenteral antibiotics, manual removal of placenta and manual vacuum aspiration/dilation and curettage. The reason for not having performed signal functions was the lack of trained staff. In fact, required health workers (gynaecologist obstetrician) are not recruited to work for these facilities.

**Table 2:** facilities by EmOC signal functions

	University maternity hospital (n=1)	Peripheral maternity facilities (n=4)
**Services provided within the past 3 months (EmOC signal functions)**		
1. Parental antibiotics	1	0/4
2. Parental oxytocics	1	4/4
3. Parental sedatives/anticonvulsants	1	4/4
4. Manual removal of placenta	1	4/4
5. Removal of retained products	1	0/4
6. Assisted vaginal delivery	1	0/4
7. Neonatal resuscitation with bag and mask	1	4/4
8. Blood transfusion provided	1	0/4
9. Caesarean section	1	0/4
Current EmOC Status		
Comprehensive EmOC	1	0
Non EmOC	0	4

**Table 3:** emergency obstetric care (EmOC) indicators

Indicator		Minimum acceptable	Value
1&2	Availability and geographical distribution of EmOC facilities	At least 5 EmOC facilities for 500 000 inhabitants including at least 1 comprehensive EmOC	0.72
3	Proportion of all births performed in EmOC facilities	To set by country	EmOC facilities : 75%
			All facilities : 95%
4	Met need for EmOC	100%	EmOC facilities : 89.5%
			All facilities: 96.8%
5	Proportion of births performed by caesarean delivery	5-15%	24.2%
6	Direct complication case fatality rate	< 1%	EmOC facilities : 0.159%
			All facilities : 0.147%
7	Intrapartum and very early neonatal death rate	To set by country	EmOC facilities : 0.65%
			All facilities : 0.51%
8	Proportion of deaths due to indirect causes	None set	0

**Utilization of EmOC services:** utilization of services was estimated through indicator 3 (proportion of all deliveries performed in fully functioning EmOC facilities), indicator 4 (met need for EmOC), and indicator 5 (proportion of deliveries performed by caesarean section) (Table 3). Overall, 14021 births were expected during the study period. Among them, 75% occurred in EmOC facilities (University hospital). In line with the number of expected births, 2103.15 direct obstetric complications were expected during the period of the study. So the met need for EmOC (total complications managed in an EmOC facility as a proportion of expected complicated deliveries in a given population) in the study area was 89.5% (Table 3). Among all births, 3397 births were performed by caesarean section in the unique CEmOC facilities. Thus, the percentage of deliveries performed by caesarean section was 24.2% (Table 3).

**Quality of EmOC services:** in total, 2036 direct obstetric complications were recorded in all the sampled facilities (Table 4). The most frequent direct reported obstetric complications are: 839 (41.21%) obstructed/prolonged labour, 397 (19.5%) pre-eclampsia/eclampsia and 309 (15.18%) haemorrhage (Table 5). During the study period, three maternal deaths from direct obstetric complications occurred. There was no maternal death from indirect obstetric complications (Table 4). The direct obstetric case fatality rate in the EmOC facilities was 0.159%, which do not exceed the UN-recommended maximum of less than 1% (Table 3). Overall, 69 intrapartum and very early neonatal deaths were recorded in all facilities (all of them in the CEmOC facility: University hospital). Intrapartum and very early neonatal death rate in EmOC facilities was 0.65% (Table 3 and Table 4).

**Table 4:** deliveries, obstetric and neonatal complications in visited facilities of the governorate of Sousse

	University Maternity Hospital (n=1)	Peripheral Maternity facilities (n=4)	All facilities
Current EmOC Status	CEmOC	Non EmOC	-
Population of Sousse governorate	-	-	690700
Crude Birth Rate of Sousse governorate	-	-	20.3/1000 inh
Expected births	-	-	14021
Expected number of direct complications	-	-	2103.15
Births	10583	2844	13427
Number of direct complications	1883	153	2036
Cesarean delivery	3379	-	3379
Maternal death from direct obstetric complications	3	0	3
Maternal death from indirect obstetric complications	0	0	0
Intrapartum and very early neonatal death	69	0	69

**Table 5:** obstetric complications treated in visited health facilities

	University Maternity Hospital(n=1)	Peripheral Maternity facilities (n=4)	All facilities
Current EmOC Status	CEmOC	Non EmOC	-
Prepartum/postpartum haemorrhage	285	24	309
Obstructed/prolonged labour	771	68	839
Ruptured uterus	72	0	72
Postpartum sepsis	219	0	219
Severe pre-eclampsia/eclampsia	336	61	397
Complications of abortion	19	0	19
Ectopic pregnancy	188	0	188

## Discussion

Some limitations may affect the generalization of the present results. In fact, only public sector facilities were studied, although some private ones could perform EmOC services in Sousse. However, the total number of births in Sousse in 2016 was 16599 among them 13427 in public sector facilities (80.9% of all births). Our findings can nonetheless provide strong insights into the state of EmOC services in the region. There is however a need for further assessments on EmOC services in Tunisia at different levels of health units and in different governorates especially because of existence of regional disparities. The minimum requirement of five EmOC facilities with at least one CEmOC facility per 500000 inhabitants was not met in our region. Inadequacy in the number of EmOC facilities has been found in many EmOC surveys [11-16]. However, data from other studies [13,14,17,18] on the availability of EmOC facilities show a tendency for countries to have an adequate number of CEmOC facilities and an inadequate number of BEmOC facilities. In 2006, in an analysis of 24 EmOC needs assessment at the national or quasi-national level, it was demonstrated that all of the countries, except two, met the minimum acceptable level of one CEmOC facility per 500000 inhabitants, including some countries with very high maternal mortality. However, in these same countries with high MMR, few basic functional facilities have been identified [15,19,20]. According to Paxton *et al*. the low proportion of BEmOC facilities results from many factors: prioritization by governments of resources for central urban hospitals at the expense of lower level facilities, difficulty of maintaining equipment and supplies in rural regions and difficulty in retaining qualified staff in smaller facilities. In addition, government regulations and policies often make it difficult for a facility without a permanent physician to perform certain signal functions [15,16].

Interventions which aim to increase the number of available BEmOC facilities and to improve the services at existing facilities are essential. In fact, most obstetric complications can be managed at the basic level and BEmOC facilities are generally more easily accessible than CEmOC centres [11-13]. Consequently, many maternal deaths can be avoided if skills to perform assisted vaginal deliveries and removal of retained products are available in peripheral maternity facilities [13-15,17,18]. None of these two functions was performed at our peripheral hospitals. This finding was reported in other countries, especially in African ones where the low rate of assisted vaginal delivery could have resulted in an increase in the caesarean delivery rate [15,21]. In Tunisia, performing assisted vaginal delivery using vacuum extraction or forceps is carried out only by obstetricians. General doctors and midwives are not authorized to perform it. Because of this criterion, many facilities could not be classified as BEmOC [10]. Management issues were the leading reasons for not performing blood transfusions and caesarean sections because generally peripheral maternity facilities in Tunisia were not equipped with operating rooms and transfusion centres [22].

To solve these problems, some countries have adopted innovative strategies, such as hiring private providers for public facilities to maximize the use of the existing public sector infrastructure [23]. A critical review of national policies and negotiation with professional organizations to delegate the function to the mid-level providers with necessary supervision, is needed and should be discussed [24]. Finally, rather than building new sophisticated hospitals, we have to upgrade poorly functioning hospitals in order to provide basic and accessible EmOC services. By doing so, the workload in central hospitals could be relieved and clinicians there could have more time to update their knowledge and provide a better quality of CEmOC [19,25]. Although the WHO handbook on monitoring emergency obstetric care recommends 5-15% of pregnancies are expected to require caesarean delivery, what matters most is all women who need this service should have it. The high caesarean section rate found in our study (24.2%) is also worrying. It needs careful interpretation and further investigations on the appropriateness of caesarean sections´ indications. This high rate may even be underestimated because the private sector was not included in our study. This finding is different from what is observed in low and middle income countries where habitually the caesarean rate remains low [17,18,20,26]. In these countries, this fact was explained on one hand by the unequal distribution of CEmOC health facilities, particularly in rural areas, and on the other hand by the low availability of qualified health workers and the insufficiency of operating theatres in district hospitals [20].

We can consider the percentage of all performed births in EmOC facilities acceptable (75%) because if all facilities are included, this proportion can reach 95%. In Tunisia, this situation is totally different from other countries where it was very low (under the 15% minimum acceptable level). This was explained by: population dispersion, poor infrastructure, lack of reliable transportation resulting in poor geographical accessibility to facilities [17,26], lack of knowledge/awareness, poor perceived service quality, lack of confidentiality and misbehaviour of health workers [26]. In our study, the met need for EmOC was 89.5% (under the 100% recommended level). However, it is higher than those reported in many African and Asian countries where this rate is sometimes under 10% indicating a poor utilization of health facilities for maternity services. A recent review has found that the met need for EmOC is negatively correlated with maternal mortality [27]; further highlighting the critical role of EmOC in maternal mortality reduction. These assessments looked mainly at the use of EmOC services but they do not provide enough information to elucidate why women use or don't use these services. In future assessments, it will be useful to look deeper into factors that affect access and use of EmOC services [24]. The direct obstetric case fatality rate, which reflects the quality of care, was 0.159% in the EmOC facilities. It did not exceed the UN-recommended maximum of less than 1%. In the area study, three maternal deaths were reported during the study period. However, in Tunisia, the MMR is still relatively high. It is estimated to be 44.8/100 000 live births [7]. In our study, the intrapartum and very early neonatal death rate, was 0.65%. There were no UN benchmarks proposed in the WHO handbook. This low rate could be explained on one hand by the existence of neonatal intensive care unit and staff and on the other hand by the sufficiency of the surveillance of delivery performance, particularly the obligation to use partogram.

## Conclusion

Institutionalizing use of EmOC process indicators will be a key step in providing regular data for national and regional analysis and planning. An important objective is that managers and heads of facilities have to be involved and take an active role in reporting and interpreting the indicators, and take measures to improve their local situation. The challenge is to ensure the availability of basic and comprehensive EmOC services 24/7 without geographic disparities. Upgrading maternities to at least basic EmOC status would be a major contributing step and will decrease the load on the University hospital.

### What is known about this topic

- Emergency obstetric care (EmOC) is a high impact priority intervention highly recommended for improving maternal health outcomes;
- Emergency obstetric care (EmOC) indicators has been used in many countries to estimate the availability, accessibility, utilization, and quality of EmOC services, to monitor and evaluate the impact of interventions improving maternal and child health outcomes.

### What this study adds

- It is the first study in Tunisia to estimate the availability, accessibility, utilization, and quality of EmOC services;
- We have to upgrade maternities to at least basic EmOC status;
- Institutionalizing the use of EmOC process indicators in our country is a necessity in order to provide regular data for national and regional analysis and planning.
